# Whole-Genome/Exome Sequencing Uncovers Mutations and Copy Number Variations in Primary Diffuse Large B-Cell Lymphoma of the Central Nervous System

**DOI:** 10.3389/fgene.2022.878618

**Published:** 2022-05-12

**Authors:** Qiong Zhu, Jianchao Wang, Wenfang Zhang, Weifeng Zhu, Zaizeng Wu, Yanping Chen, Musheng Chen, Limei Zheng, Jianqing Tang, Sheng Zhang, Di Wang, Xingfu Wang, Gang Chen

**Affiliations:** ^1^ Department of Molecular Pathology, Fujian Cancer Hospital, Fujian Medical University Cancer Hospital, Fuzhou, China; ^2^ Department of Pathology, Fujian Cancer Hospital, Fujian Medical University Cancer Hospital, Fuzhou, China; ^3^ The School of Basic Medical Sciences, Fujian Medical University, Fuzhou, China; ^4^ Department of Pathology, The First Affiliated Hospital of Fujian Medical University, Fuzhou, China

**Keywords:** mutation, copy number variations, KMT2D, 1q31.3 amplification, prognosis

## Abstract

**Background/objective:** Identification of key genetic alterations is of importance in the targeted therapies of primary central nervous system lymphoma (PCNSL). However, only a small number of studies have been carried out in PCNSL. In this study, we further described the genetic mutations and copy number variations (CNVs) in PCNSL patients using whole-genome/exome sequencing (WGS/WES), as well as revealed their associations with patients’ clinicopathological features and prognosis.

**Methods:** Tumor specimens from 38 patients with primary diffuse large B-cell lymphoma of the central nervous system (CNS DLBCL) were enrolled to WGS (*n* = 24) or WES (*n* = 14). The CNVs and mutations of 24 samples (WGS) and 38 samples (WGS/WES) were characterized, respectively. The associations between CNVs and mutations with the overall survival rates of PCNSL patients were also evaluated.

**Results:** The most common mutations were identified in *IGLL5* (68%), *PIM1* (63%), *MYD88* (55%), *CD79B* (42%), *BTG2* (39%), *PCLO* (39%), *KMT2D* (34%), and *BTG1* (29%) genes. Among the mutated genes, *EP300*, *ETV6,* and *HIST1H1E* mutations were exclusively detected in the elderly, while *DUSP2* mutations were associated with the immune microenvironment indicators. In addition, *KMT2D* mutation was associated with a poor prognosis. In addition, 488 CNVs including 91 gains and 397 deletions were observed across 24 samples from WGS results. Notably, 1q31.3 amplification was closely associated with the poor prognosis of PCNSL patients.

**Conclusion:** This study further characterizes the genomic landscape of primary CNS DLBCL using WGS/WES, which provides insight into understanding the pathogenesis of PCNSL and fosters new ideas for the targeted treatment of PCNSL.

## Introduction

Primary central nervous system lymphoma (PCNSL) is a rare extranodal non-Hodgkin’s lymphoma (NHL), with high aggressiveness and poor outcome. More than 95% of PCNSL cases are diffuse large B-cell lymphoma (DLBCL) (Montesinos-Rongen et al., 2008; Citterio et al., 2017; Grommes and DeAngelis 2017), which is divided into germinal center B-cell like (GCB), activated B-cell like (ABC), and unclassifiable subtypes according to cell-of-origin (COO). Central nervous system (CNS) DLBCL accounts for 2.4–3% of all primary brain tumors and less than 1% of NHL in adults, with increasing incidence year by year, especially in the elderly (Ricard et al., 2012; O'Neill et al., 2013). The methotrexate-based regimen is the common chemotherapy method for patients with PCNSL, which improves patients’ outcomes but also brings toxicities inevitably, such as hepatotoxicity, neurotoxicity, myelotoxicity, and mucositis, and the long-term survival rate of PCNSL remains low (Howard et al., 2016; Royer-Perron and Hoang-Xuan 2018). Therefore, it is necessary to comprehensively reveal the genetic alterations underlying PCNSL and offer new therapeutic targets.

With the development of cutting-edge, high-throughput molecular techniques in formalin-fixed and paraffin-embedded (FFPE) specimens, several recurrent mutations, and copy number variability (CNV) have been identified in PCNSL, including the mutations in *PIM1* (41.4–100%) (Fukumura et al., 2016; Zhou et al., 2022), *BTG2* (12.5–92.7%) (Fukumura et al., 2016; Zhou et al., 2022), *MYD88* (38–85.4%) (Montesinos-Rongen et al., 2011; Gonzalez-Aguilar et al., 2012; Braggio et al., 2015; Fukumura et al., 2016; Zhou et al., 2022), *CD79B* (51.2%) (Zhou et al., 2022), *CDKN2A* (60%) (Braggio et al., 2015), *PRDM1* (19%) (Courts et al., 2008), *CARD11* (16%) (Montesinos-Rongen et al., 2010), and *TBL1XR1* (14%) (Gonzalez-Aguilar et al., 2012) genes and the common CNVs of deletions of 6q (55%) and 6p21 (50%), and gains of 7q (39%) and 11q (28%) (Booman et al., 2008; Schwindt et al., 2009; Braggio et al., 2011). Noticeably, evidence has shown that genetic alterations are closely associated with the prognosis of patients with PCNSL. For example, Fukumura et al. (2016) reported that the focal deletion or somatic mutations of *HLA* genes were linked to poor prognosis. Zhou et al. (2022) recently demonstrated that patients with *CD79B* and/or *PIM1* mutations had a significantly longer 2-year overall survival (OS) (76 and 40%, *p* = 0.0372) than those without *CD79B* or *PIM1* mutations. These findings indicate the importance of the detection of genetic mutations in PCNSL. Regrettably, only a small number of genetic studies have been carried out in PCNSL due to the rarity of PCNSL and the difficulty in acquisition of the intracranial samples, so further analysis is crucial.

In this study, we aimed to further characterize the mutation and CNV profiles of PCNSL using whole-genome sequencing (WGS) and whole-exome sequencing (WES), as well as to reveal their associations with patients’ clinicopathologic features and prognosis.

## Patients and Methods

### Clinical Samples

Brain specimens of 38 patients diagnosed with primary CNS DLBCL from Fujian Cancer Hospital and The First Affiliated Hospital of Fujian Medical University between February 2012 and October 2020 were included in this study. All cases were newly diagnosed primary CNS DLBCL (Ferreri, 2011), and the samples were sequenced successfully. No apparent immunodeficiency was observed in all of the patients, and all cases were followed up to 31 July 2021. The general clinical characteristics, including age, sex, COO, treatment methods, OS, and immunohistochemical indexes, were shown in Supplementary Table S1. Experiments involving clinical human samples were conducted referring to the Helsinki Declaration, and this study was approved by the Institutional Ethical Standards of Committee at Fujian Cancer Hospital (Approval no. SQ 2020-106-01). The informed consent was obtained from every patient.

### WGS and WES

Genomic DNA (gDNA) was extracted from the brain tissues of 38 patients with primary CNS DLBCL using the FFPE tissue kit (No. 56404, Qiagen, NV, Venlo, Netherlands) according to the manufacturer’s instructions. Then, gDNA was digested into ∼200 bp fragments using a focused ultrasonicator (No. M220, Covaris, Woburn, MA, United States). After quality control and equimolar pooling, WGS was carried out using DNBSEQ-T7 sequencing instruments (MGI, Guangdong, China). Meanwhile, WES was performed on Illumina NovaSeq 6,000 sequencing instruments (Illumina, San Diego, CA, United States). The quality of raw sequencing data was evaluated using the FastQC (version 1.11.4) software. In addition, the raw sequencing data were then processed through the use of Trimmomatic (version 3.6) software to remove sequencing adapters and low-quality reads referring to the joint sequence fragments of the 3′ end and low-quality fragments with Q value <25, as well as the fragments with <35 bp. To prepare read alignments for analysis, we processed all sequence data through the Broad Institute’s data processing pipeline. Reads were aligned to the Human Genome Reference Consortium build 37 (GRCh37) using BWA (version 0.5.9-tpx). These bam files contain reads aligned to the human genome with quality scores recalibrated using the TableRecalibration tool from the Genome Analysis Toolkit (GATK) (version 4.1.4.0). Variant detection and analysis of the BAM files were then performed using the Broad Institute’s Cancer Genome Analysis infrastructure program Mutect2 (Cibulskis et al., 2013; Boutros et al., 2014). Mutations were finally annotated using the Annovar software (version 2017-07-17).

### Mutation Analysis and Validation

Variants were screened by Shanghai Rightongene Biotechnology Co., Ltd. (Shanghai, China) based on the filtering conditions: 1) SNPs (single nucleotide polymorphisms) or Indels (insertions and deletions) with a mutation allele frequency (MAF) ≥ 0.001 in databases of 1,000 genomes project (Abecasis et al., 2010), 1000 genome East Asian, ExAC all or ExAC East Asian, and genomAD (Zou et al., 2016) were removed; 2) mutation sites are functionally annotated in the KEGG (Kyoto Encyclopedia of Genes and Genomes) database were retained; 3) dbSNP (v147) sites in the COSMIC database were retained; 4) SNPs or Indels including stop-gain, stop-loss, frameshift, non-frameshift, splicing sites, and missense were retained; 5) SNPs or Indels detected in the full genetic database for cancer (MSK-Impact, Foundation One) or DLBCL-related gene list (Supplementary Table S2) were reserved. “Maftools” package (version 2.2.10) of the R software (Mayakonda et al., 2018) was used to draw the horizontal histogram which shows genes with a higher mutation frequency in the 38 cases, as well as to demonstrate the mutation sites of *KMT2D* gene in its domain region. Mutations in some genes including *MYD88*, *CD79B*, *KMT2D*, *CDKN2A,* and *PIM1* with VAF ≥10% were validated by Sanger sequencing. Mutations verified and primers used in the Sanger sequencing are listed in Supplementary Table S3.

### CNV Profiles

The CNV data of 24 brain tissues that received WGS were processed using the software package CNVkit (Talevich et al., 2016) and Nexus software version 5 (Biodiscovery, El Segundo, CA). Then, the CNV analysis was performed using GISTIC 2.0.23 software (Beroukhim et al., 2007). To identify whether amplified or deleted regions within a chromosome were statistically significant, GISTIC was conducted by setting the q-value threshold of 0.1. Focal amplification or deletion for GRCh37 was determined by setting the broad length cutoff to 0.5 and the confidence level to 0.9. Meanwhile, the other parameters are restricted to their default values.

### Functional Enrichment Analysis

Gene ontology (GO) analysis, covering biological processes, cellular components, and molecular functions, was used to evaluate the enriched functions of the genes identified from the CNV dysregulation using DAVID (https://david.ncifcrf.gov/). Fisher’s exact test was then applied to detect overlapping genes in our WGS/WES and the GO annotation list beyond that which would be expected by chance. The Kyoto Encyclopedia of Genes and Genomes (KEGG) database was used to understand the high-level functions and effects of the biological system (http://www.genome.jp/kegg/). DAVID was also applied to analyze the KEGG pathway that enriched genes with CNV. The *p*-value < 0.05 was considered to be significantly enriched.

### Statistical Analysis

The maftools (“clinicalEnrichment”) of the R packages was employed to examine the relationship between the mutation profiles and age (<60, ≥60), LDH (lactate dehydrogenase) level (elevated, normal), COO (GCB, non-GCB), c-MYC expression (<40, ≥40%), BCL2 expression (<50%, ≥50%), BCL6 (positive, negative; cut off = 30%) (Hans et al., 2004), P53 (<50%, ≥50%), pSTAT (<40, ≥40%), proportion of infiltrated T cells (<30%, ≥30%), CD8 cells/CD3 cells (%) (<average, ≥ average), proportion of PD-L1-positive cells CPS (<10%, ≥10%), proportion of CD163-positive cells (CD163^+^ cell number/total cell number, < 30%, ≥30%) (Alame et al., 2020), and the number of FOXP3-positive T cells (2 mm^2^) (<average, ≥ average) of PCNSL patients using multiple hypothesis testing to assess the false discovery rate (q-value). Kaplan-Meier (K-M) curves with log-rank tests were used to analyze the clinical value of CNVs and mutations in the OS of patients with PCNSL. *p*-value or adjusted *p*-value < 0.05 (q-value) was thought to be a significant difference.

## Results

### Baseline Characteristics of the 38 Patients With Primary CNS DLBCL

A total of 89 samples were collected for sequencing, but 51 samples were excluded due to the low content of gDNA or unqualified sequencing data, and the other 38 samples were sequenced successfully and included in this study. Among the 38 patients, 52.63% (20/38) cases were male and 47.36% (18/38) were female, and 7 cases (18.4%) were GCB and 31 (81.6%) cases were non-GCB defined according to the COO (Hans et al., 2004). The age of patients ranged from 41 to 77 years, with 17 patients <60 years and 21 patients ≥60 years. Ten patients underwent surgery, 20 patients received surgery and chemotherapy, six patients were given surgery, chemotherapy, and radiotherapy, and two patients underwent surgery and radiotherapy after the diagnosis of primary CNS DLBCL. Detailed information about the 38 cases with primary CNS DLBCL is summarized in Table 1 and Supplementary Table S1.

**TABLE 1:** Clinicopathological information of the 38 PCNSL patients.

Factor	n (%)
Age	
<60, years	17 (44.7)
≥60, years	21 (55.3)
Sex	
Male	20 (52.6)
Female	18 (47.4)
COO	
GCB	7 (18.4)
Non-GCB	31 (81.6)
LDH	
High	6 (15.8)
Elevated	26 (68.4)
NA	6 (15.8)
Treatment method	
Surgery	10 (26.3)
Surgery + chemotherapy	20 (52.6)
Surgery + radiotherapy	2 (5.3)
Surgery + chemotherapy + radiotherapy	6 (15.8)

NA, not available.

### Mutation Profiles of the 38 Patients With Primary CNS DLBCL

**FIGURE 1:**
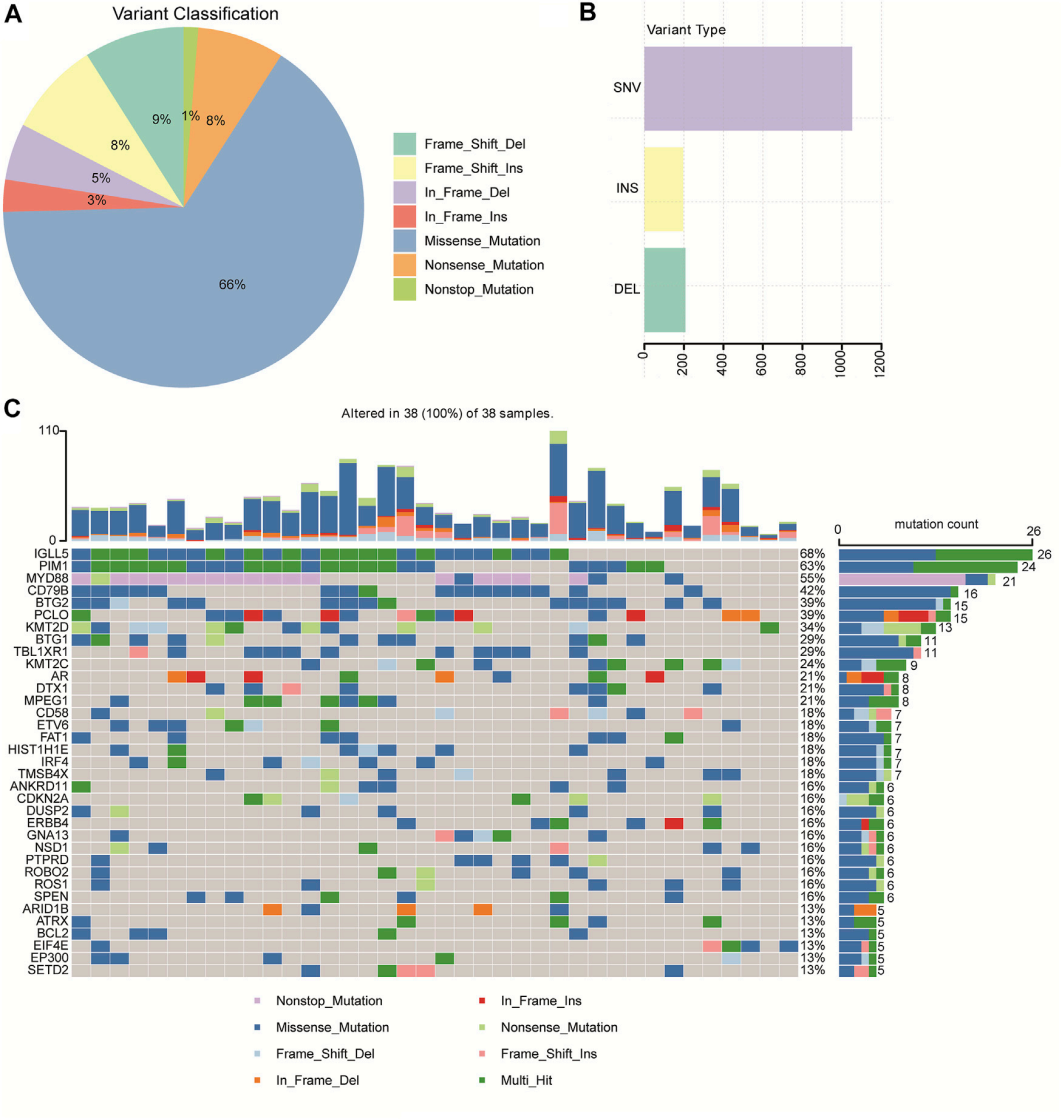
Mutation profiles identified by WGS/WES in 38 patients with primary CNS DLBCL. **(A)** Pie chart showing the percentages of different types of mutation in primary CNS DLBCL. **(B)** Number of SNV (single nucleotide variation), INS (insert), and DEL (deletion) detected in primary CNS DLBCL. **(C)** Genes with the most frequent mutations in each patient with primary CNS DLBCL.

A total of 1,456 mutation sites (335 genes), including SNVs and INDELs, from the 38 brain tissues of primary CNS DLBCL patients were identified. The most commonly detected mutations were missense mutation, which was ∼7 times the second mutation type (frame shift deletion) (Figure 1A). In the 38 cases, SNV occurred more frequently (about 5-fold) than the insertion and deletion types (Figure 1B). The top ten most frequently mutated genes were *IGLL5* (68%), *PIM1* (63%), *MYD88* (55%), *CD79B* (42%), *BTG2* (39%), *PCLO* (39%), *KMT2D* (34%), *BTG1* (29%), *TBL1XR1* (29%), and *KMT2C* (24%) (Figure 1C). The mutation genes identified in our cohort were then compared to other WES-based studies, including Vater et al. (2015), Bruno et al. (2014), Fukumura et al. (2016), and Takashima et al. (2018). When taking into account these four cohorts, mutations in 226 genes were exclusively found in “our cohort” (Supplementary Figure S1). Two genes, *PIM1*, the cell cycle/adhesion gene (Bodor et al., 2020; Ou et al., 2020), and *MYD88*, that were detected in our cohort have also been reported in multiple studies, including Vater et al. (2015), Bruno et al. (2014), Fukumura et al. (2016), and Takashima et al. (2018). Among these 226 genes identified exclusively in our cohort, 28 genes were detected in at least 4 cases, including *CXCR4, RECQL, MSH3, RAD51B, RBM10, MAP3K13, KDM5A, EPHA5, RHOA, KMT2B, GNA13, DROSHA, EIF4E, PLK2, LYN, ZFP36L1, CHD2, ITPKB, SF3B1, RAD54L, SHOC2, PGR, POLE, ROBO2, HIST1H2BD, CDKN2A, HIST1H1C,* and *KMT2C*.

In addition, we performed GO and KEGG analysis to identify the enriched pathway of the 335 mutation genes. GO analysis revealed a significant enrichment of the mutated genes in peptidyl−tyrosine phosphorylation, positive regulation of transcription from RNA polymerase II promoter, transmembrane receptor protein tyrosine kinase signaling pathway, positive regulation of cell proliferation, negative regulation of apoptotic process, protein binding, and transmembrane receptor protein tyrosine kinase activity nucleoplasm pathways (Supplementary Figure S2A). The KEGG analysis showed that the 335 mutated genes were enriched in the B cell receptor signaling pathway, the PI3K−Akt signaling pathway, and pathways related to cancer (Supplementary Figure S2B).

### Associations of Mutations With Patients’ Clinicopathological Characteristics

**FIGURE 2:**
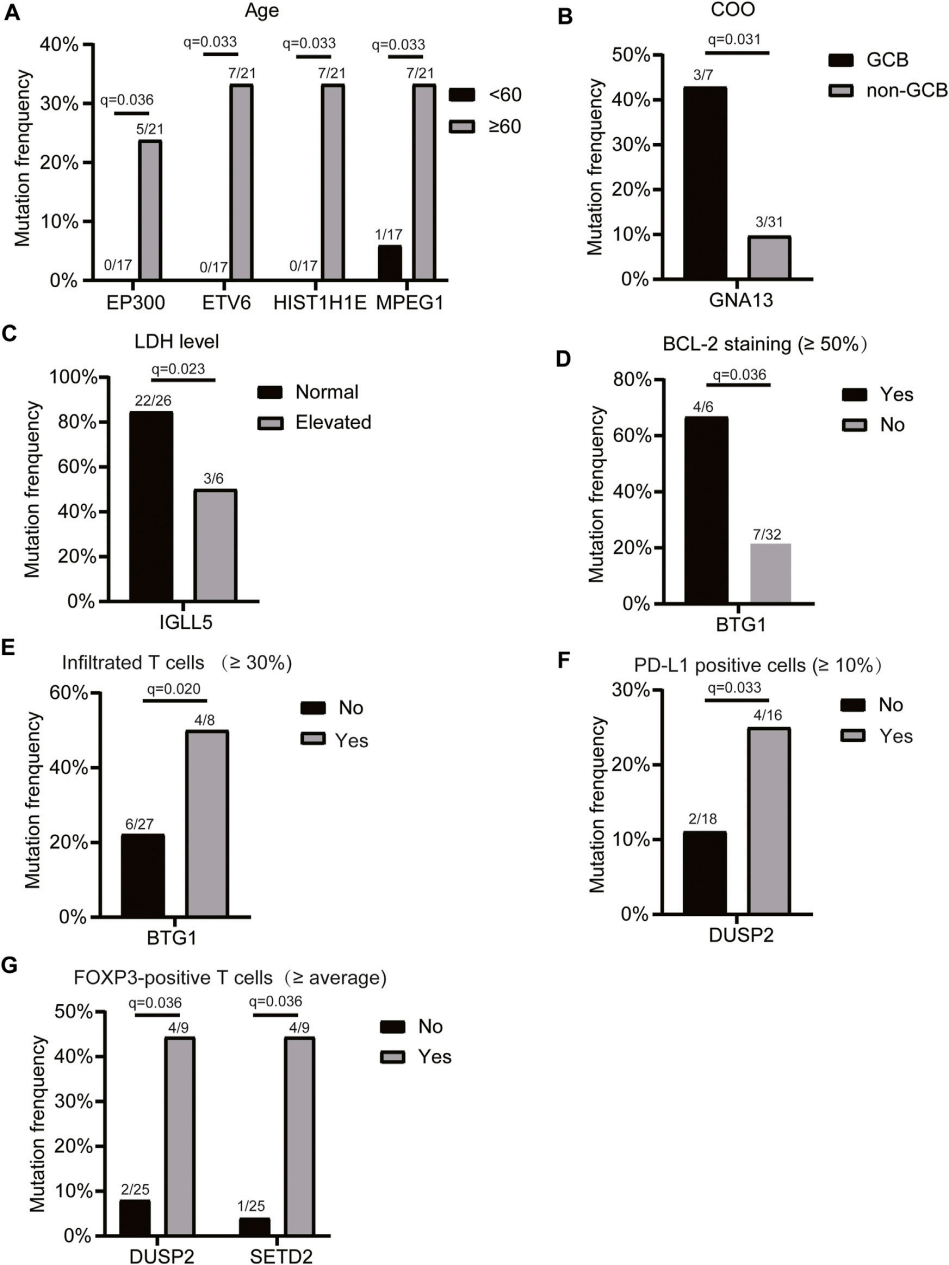
Relationship between the mutation profiles and clinicopathologic features of patients with primary CNS DLBCL. Genes with significantly different mutation frequencies in primary CNS DLBCL patients with different **(A)** ages, **(B)** COO classifications, **(C)** LDH levels, **(D)** BCL2 staining, **(E)** infiltrated T cell populations, **(F)** PD-L1-positive cell populations, and **(G)** FOXP3-positive T cell populations. The mutation count/total count and q-value were shown in the histograms.

Next, we assessed the relationship between the mutation profiles and patients’ clinicopathologic features, including age, LDH level, COO, c-MYC expression, BCL2 expression, BCL6, P53, pSTAT3, CD8/CD3 population, proportion of infiltrated T cells, CD8 cells/CD3 cells (%), proportion of PD-L1-positive cells, proportion of CD163-positive cells, and the number of FOXP3-positive T cells (2 mm^2^). The results showed that the mutations of *EP300, ETV6, and HIST1H1E* genes were only detected in the elderly (Figure 2A). The *GNA13* mutation frequency was significantly higher in the GCB subtype than that of the non-GCB group (Figure 2B). *IGLL5* mutation frequency was significantly lower in patients with elevated LDH levels (Figure 2C), while *BTG1* mutation was associated with enhanced BCL2 staining (Figure 2D), and a higher infiltrated T cell population (Figure 2E). In addition, we found that the *DUSP2* mutation was associated with a higher PD-L1-positive cell population (Figure 2F). Also, DUSP2 and SETD2 mutations were related to the higher FOXP3-positive cell population (Figure 2G). Taken together, these findings suggested that the mutated genes were associated with the clinicopathological characteristics of patients with primary CNS DLBCL.

### *KMT2D* Mutation Was Associated With Lower OS Rate in Patients With Primary CNS DLBCL

**FIGURE 3:**
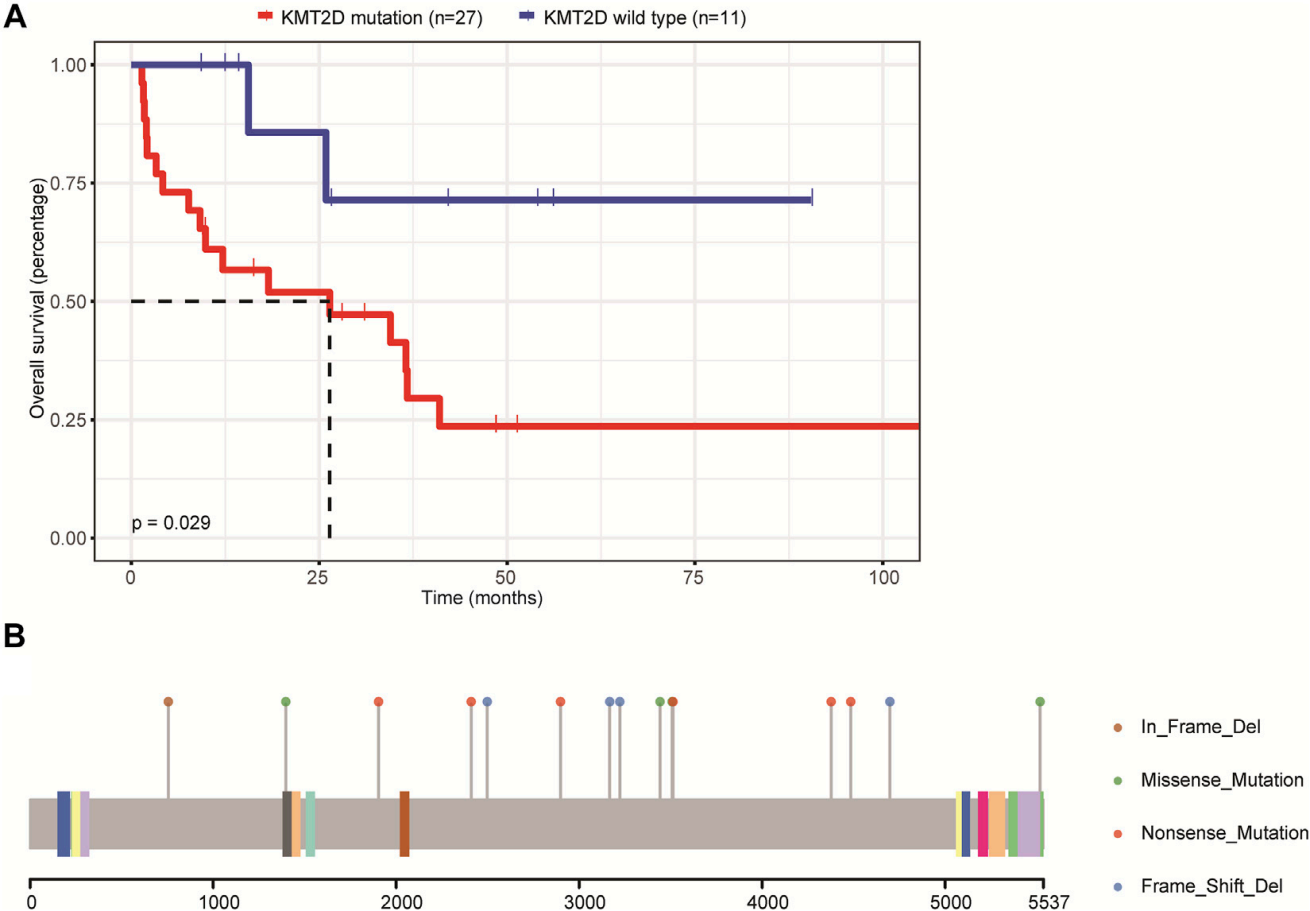
The relationship between mutations and the prognosis of patients with primary CNS DLBCL. **(A)** The K-M curve showed KMT2D mutation was linked to lower OS. **(B)** The mutation schematic diagram of the KMT2D gene in its domain region.

We then assessed the relationship between the mutations and patients’ OS. Due to the small sample size included in this study, we only focused on genes whose mutations were found in at least eight patients, including *IGLL5*, *PIM1*, *MYD88*, *CD79B*, *BTG2*, *PCLO*, *KMT2D*, *BTG1*, *TBL1XR1*, *KMT2C*, *AR*, *DTX1,* and *MPEG1*. The results demonstrated that only the mutations in the *KMT2D* gene were associated with patients’ OS and that the OS was significantly decreased in patients carrying *KMT2D* mutation as compared to patients with the wild type of *KMT2D* (Figure 3A). Figure 3B illustrates the mutation schematic diagram of the *KMT2D* gene in its domain region. These results demonstrated that *KMT2D* might be a promising marker for prognosis assessment in patients with primary CNS DLBCL.

### CNV Profiles of the 24 Patients With Primary CNS DLBCL

**FIGURE 4:**
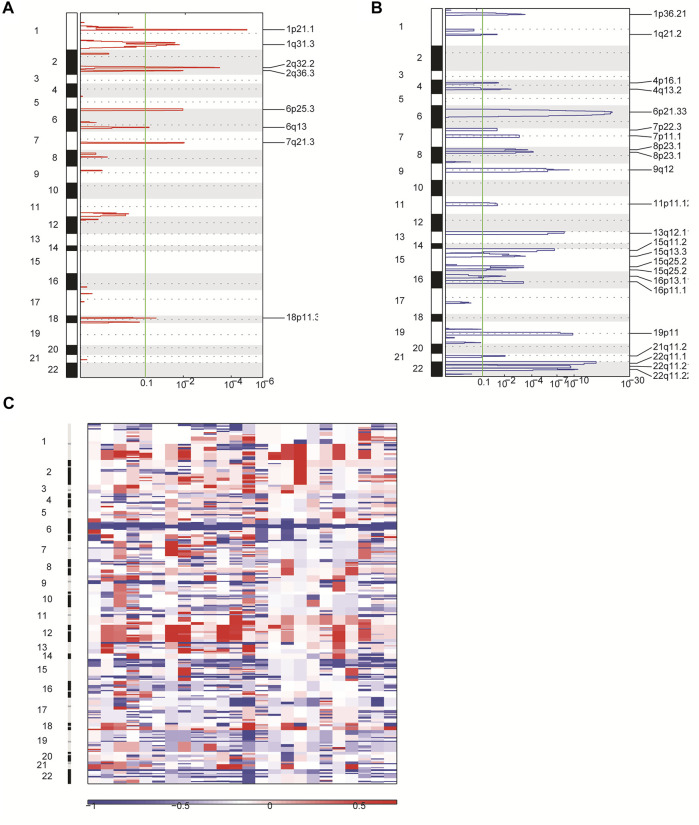
Overview of CNVs in 24 patients with primary CNS DLBCL using WGS. **(A,B)** CNVs were analyzed by GISTIC. Chromosome positions are indicated along the y axis. On the x-axis, focal amplifications **(A)** or deletions **(B)** are marked with horizontal blue or red bars, respectively. The green line represents the significance threshold of q < 0.1 (the false discovery rate after multiple hypothesis testing). **(C)** Heat map images of the 24 patients with primary CNS DLBCL. In each heat map, the samples are arranged from left to right, and the chromosome arrangement flows vertical, top to bottom ordering. Red represents CN gain and blue represents CN loss.

In order to identify other genetic abnormalities in primary CNS DLBCL, we assessed the CNV profiles of primary CNS DLBCL in 24 cases submitted to WGS. All of the detected 24 patients with primary CNS DLBCL showed a complex genome with a median of 21 CNVs per patient (range 7–30). Overall, 488 CNVs were detected across the 24 samples, including 91 gains and 397 deletions. Deletions were detected in all of the 24 patients, while gains were detected in 22 patients. A total of 31 significant variants were identified across the 24 cases at a false discovery rate of q < 0.1, including eight amplifications (Figure 4A) and 23 deletions (Figure 4B). Among them, 1q31.3, 2q32.2, and 2q36.3 were the most common amplifications, which were found in greater than 13 cases, and 6p21.33, 8p23.1, 22q11.1, and 22q11.21 were the most common deletions, which were detected in at least 20 cases (Figure 4C).

**FIGURE 5:**
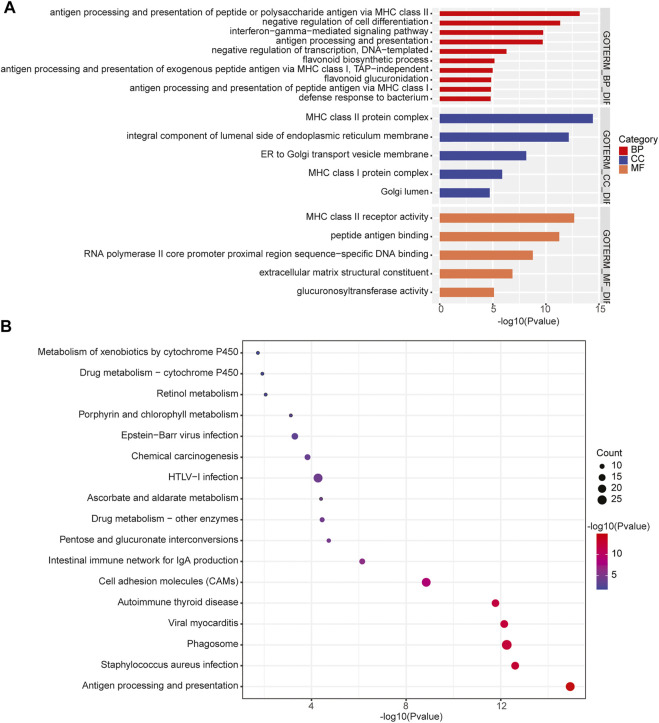
Enrichment of the CNV-related genes identified in primary CNS DLBCL. **(A)** GO and **(B)** KEGG analysis of the enriched pathways of the 1,208 genes located in the detected CNV regions.

In addition, we performed the GO enrichment and KEGG pathway analysis to assess the function of the 1,208 genes identified in 31 significant CNV segments. The GO analysis demonstrated that these genes were involved in MHC class II receptor activity, peptide antigen binding, MHC class II protein complex, antigen processing, and presentation of peptide or polysaccharide antigen via MHC class II and interferon-gamma-mediated signaling pathways (Figure 5A). The KEGG analysis showed the CNV-related genes were enriched in the cell adhesion molecules (CAMs) intestinal immune network for IgA production and antigen processing and presentation (Figure 5B). As most of the enriched pathways are related to the immune (Holling et al., 2004), we speculated that the immune pathway might play a prominent role in the occurrence of primary CNS DLBCL.

### 1q31.3 Amplification Was Associated With Lower OS Rate in Patients With Primary CNS DLBCL

**FIGURE 6:**
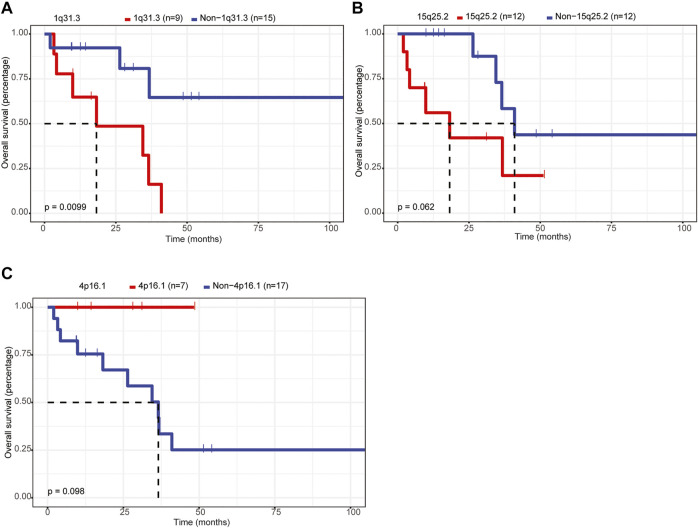
Assessment of the relationship between CNVs and OS in patients with primary CNS DLBCL. **(A–C)** K-M analysis of the relationship between the CNVs (1q31.3 amplification, 15q25.2 deletion, 4p16.1 deletion) and the OS of patients with primary CNS DLBCL.

Furthermore, we assessed the relationship between CNVs and the OS of patients with primary CNS DLBCL. Twenty CNV events, including amplifications of 1p21.1, 1q31.3, 2q32.2, 2q36.3, 6p25.3, 6q13, 7q21.3, and 18p11.31 and deletions of 1p36.21, 1q21.2, 4p16.1, 4q13.2, 7p22.3, 7p11.1, 8p23.1, 9q12, 13q12.11, 15q25.2, 16p11.1, and 21q11.2, detected in 6–18 cases were analyzed owing to the small sample size (n = 24) of the current study. The results showed that only 1q31.3 amplification was significantly associated with the prognosis of patients with primary CNS DLBCL, and it was an adverse prognostic index (Figure 6A, *p* = 0.0099). If the *p*-value was defined as 0.1, the deletion of 15q15.1 was also linked to a lower OS rate (Figure 6B), while the 4p16.1 deletion was associated with better OS (Figure 6C). These results supported the notion that CNVs were closely linked to patients’ prognosis in primary CNS DLBCL.

## Discussion

Although RNA-sequencing, high-resolution genomic arrays, and WES have been used to assess the chromosomal abnormalities and gene mutations in PCNSL (Tun et al., 2008; Braggio et al., 2011; Braggio et al., 2015; Takashima et al., 2018; Nayyar et al., 2019; Bodor et al., 2020), knowledge about the genomic profile of PCNSL is still far from enough owing to the high heterogeneity and rare clinical samples. In the present study, we employed the WES and WGS approaches to explore the genetic mutations and CNVs in patients with primary CNS DLBCL, as well as reveal their clinical values in predicting the prognosis of PCNSL.

Mutations in *IGLL5* (68%), *PIM1* (63%), *MYD88* (55%), *CD79B* (42%), *BTG2* (39%), *PCLO* (39%), *KMT2D* (34%), *BTG1* (29%), *TBL1XR1* (29%), and *KMT2C* (24%) genes were the most common mutations found in this cohort. Consistently, mutations in these genes were also detected in other PCNSL cohorts (Bruno et al., 2014; Vater et al., 2015; Fukumura et al., 2016; Takashima et al., 2018). Evidence has demonstrated that the mutation frequencies of the same genes are varying between different cohorts. For example, Montesinos-Rongen et al. (Montesinos-Rongen et al., 2011) reported that mutations of the *MYD88* gene were found in 50% (7/14) of PCNSL patients. Fukumura et al. (Fukumura et al., 2016) found *MYD88* mutations in 85.4% (35/41) of patients with PCNSL. Zhou et al. (Zhou et al., 2022) recently reported that *MYD88* mutations were detected in 60% (24/40) of Chinese patients with PCNSL. Here, 55.3% (21/38) of Chinese patients with primary CNS DLBCL presented with *MYD88* mutations. The different races, filter conditions, and high heterogeneity of the disease may cause this difference. In addition, mutations in some genes were only found in our cohort with ≥10% frequency, such as *RECQL*, *MSH3*, *RAD51B*, *RBM10, MAP3K13*, *KDM5A*, *EPHA5*, and *KMT2B.* Noticeably, mutations of these genes have been reported in B-cell lymphoma and/or to be associated with B-cell lymphomagenesis according to the COSMIC database (https://cancer.sanger.ac.uk/cosmic?genome=37). Also, the different cohorts and filter conditions as well as the high heterogeneity of PCNSL may cause this discrepancy.

Moreover, we found that the mutations of *EP300*, *ETV6*, *HIST1H1E,* and *MPEG1* genes were age-related, and all of them showed higher mutation frequencies in patients aged ≥60 years than in those aged <60 years. *EP300* is a histone acetyltransferase and functions as a transcriptional co-activator via H3K27 acetylation. Clinically, *CREBBP* and *EP300* variants are frequently reported in DLBCL patients, are often mutually exclusive, and contribute to disease relapse and inferior prognosis (Juskevicius et al., 2017). Consistently, mutations in the *CREBBP* and *EP300* genes were mutually exclusive in our cohort. It has been demonstrated that *ETV6* mutation is a predictor of shortened survival in myelodysplastic syndromes (Bejar et al., 2011). In our cohort, *ETV6* mutation was observed in 7 (18%) cases but showed no significant association with the OS of primary CNS DLBCL. It has been reported that *HIST1H1E* mutations are linked to the tumor microenvironment in DLBCL with extranodal invasion (Shen et al., 2020). Nevertheless, we did not find any association between *HIST1H1E* mutations and the immune microenvironment in PCNSL patients. Instead, *DUSP2* mutations were demonstrated to be linked to immune microenvironment indicators, such as the populations of PD-L1-positive cells and FOXP3-positive T cells.

Also, we assessed the relationship between the mutation profiles and OS in patients with primary CNS DLBCL. The results demonstrated that only the mutations of the *KMT2D* gene were associated with the poor prognosis of patients in our cohort. The gene encoding the lysine-specific histone methyltransferase KMT2D has emerged as one of the most frequently mutated genes in follicular lymphoma and DLBCL. Also, evidence has shown that *KMT2D* functions as a tumor suppressor and that its genetic ablation in B cells promotes lymphoma development *in vivo* (Ortega-Molina et al., 2015). Also, studies have demonstrated that *KMT2D* mutations were significantly associated with shorter OS in lung cancer (Ardeshir-Larijani et al., 2018), mantle cell lymphoma receiving high-dose therapy (Ferrero et al., 2020), and natural killer-cell lymphoproliferative disorders (Gao et al., 2019). Zhou et al. (Zhou et al., 2022) reported that patients with *CD79B* and/or *PIM1* mutations had a significantly longer 2-year OS (76 and 40%, *p* = 0.0372) than those without *CD79B/PIM1* mutations in primary CNS DLBCL patients. Here, we assessed the influence of *PIM1* and *CD79B* mutations on the OS of patients with primary CNS DLBCL, respectively. However, the results showed that both *PIM1* and *CD79B* mutations showed no significant association with the OS of primary CNS DLBCL patients. This may also be caused by the small sample size. In addition, we assessed the effect of *PIM1* and/or *CD79B* mutations on the 2-year survival of patients with primary CNS DLBCL. The results showed that the 2-year survival of patients without *PIM1* and/or *CD79B* mutations was 40% (2/5, with the other two patients lost to follow), which was slightly lower than that of patients with *PIM1* and/or *CD79B* mutation (48%, 15/31), and the difference showed no significance.

Moreover, we characterized the CNV profiles in 24 samples submitted to WGS. We discovered 1q31.3, 2q32.2, and 2q36.3 were the most common amplifications, and 6p21.33, 8p23.1, 22q11.1, and 22q11.21 were the most common deletions. Studies have shown that deletions of 6p21, 6q, and gains of 7q, 11q, and 12q are the common CNVs in PCNSL (Booman et al., 2008; Schwindt et al., 2009; Braggio et al., 2011). Consistently, 6p21.33 deletion was detected in all of the 24 PCNSL samples. Also, amplification in 7q21.3 was detected in 11 out of 24 patients (46%). Noticeably, we found that 1q31.3 amplification emerged as an adverse prognosis factor for PCNSL patients in our cohort. *CFH*, *KCNT2*, *MIR4735*, *CFHR1*, *CFHR3*, *CFHR4*, *CFHR2*, *CFHR5*, *ASPM*, *ATP6V1G3, C1orf53, CRB1, DENND1B, F13B, LHX9, NEK7, PTPRC,* and *ZBTB41* were the genes involved in 1q31.3. Among these genes, *CFH* (complement factor H) and its related family members, *CFHR1–5,* encoded soluble proteins which play crucial roles in immune responses (Jozsi and Zipfel, 2008; Abarrategui-Garrido et al., 2009; Bajic et al., 2015), suggesting the involvement of the immune system in PCNSL. In addition, evidence has demonstrated that *ASPM* (spindle-like microcephaly-associated protein) expression is elevated in DLBCL tissues, and the high expression of *ASPM* is correlated with aggressive clinicopathological characteristics and serves as an independent factor of poor prognosis (Wu et al., 2021). Subsequently, the *in vitro* assays confirmed that *ASPM* functioned as an oncogene in DLBCL (Wu et al., 2021), suggesting a potential role of *ASPM* in PCNSL, which should be verified in the future.

At the molecular level, DLBCL can be divided into four genetic subtypes with different outcomes, including MCD (based on the co-occurrence of *MYD88* ^*L265P*^ and *CD79B* mutations), BN2 (based on *BCL6* fusions and *NOTCH2* mutations), N1 (based on *NOTCH1* mutations), and EZB (based on *EZH2* mutations and *BCL2* translocations). According to this classification, 30–40% of primary CNS DLBCL cases are divided into the MCD subtype (Ho and Grommes, 2019; Zhou et al., 2022). Here, we found that 17 (45%) of the 38 patients were divided into the MCD subtype. Unfortunately, we observed that the molecular classification of systemic DLBCL was not applicable to primary CNS DLBCL for prognosis evaluation, as previously reported (Zhou et al., 2022).

Several limitations should be stated. The main drawback is that the sample size is too small which is mainly caused by the low incidence of primary CNS DLBCL. The other is that we did not include negative control samples for the 38 cases of primary CNS DLBCL mainly because of the high cost of WGS/WES. We intend to extend our study to further explore the genetic changes and their clinical values. Also, it is a pity that we did not explore the pathogenic role of some mutations *in vivo* and *in vitro*, and we intend to explore the role of mutations in the development of PCNSL, e.g., mutations of *KMT2D* and *CDKN2A* genes, in future studies.

## Conclusion

In summary, this study described novel genetic characteristics of primary CNS DLBCL using WGS/WES. Noticeably, *KMT2D* mutation and 1q31.3 amplification were significantly associated with the poor prognosis of patients with primary CNS DLBCL. This study provides novel insights into the knowledge underlying PCNSL pathogenesis at the genetic level.

## Data Availability

The original contributions presented in the study are included in the article/Supplementary Material, further inquiries can be directed to the corresponding authors.
