# Supplementary figures and images for: Whole-Genome/Exome Sequencing Uncovers Mutations and Copy Number Variations in Primary Diffuse Large B-Cell Lymphoma of the Central Nervous System
Source: Front Genet. 2022 May 12;13:878618. doi: 10.3389/fgene.2022.878618 (PMC9133733; doi:10.3389/fgene.2022.878618)

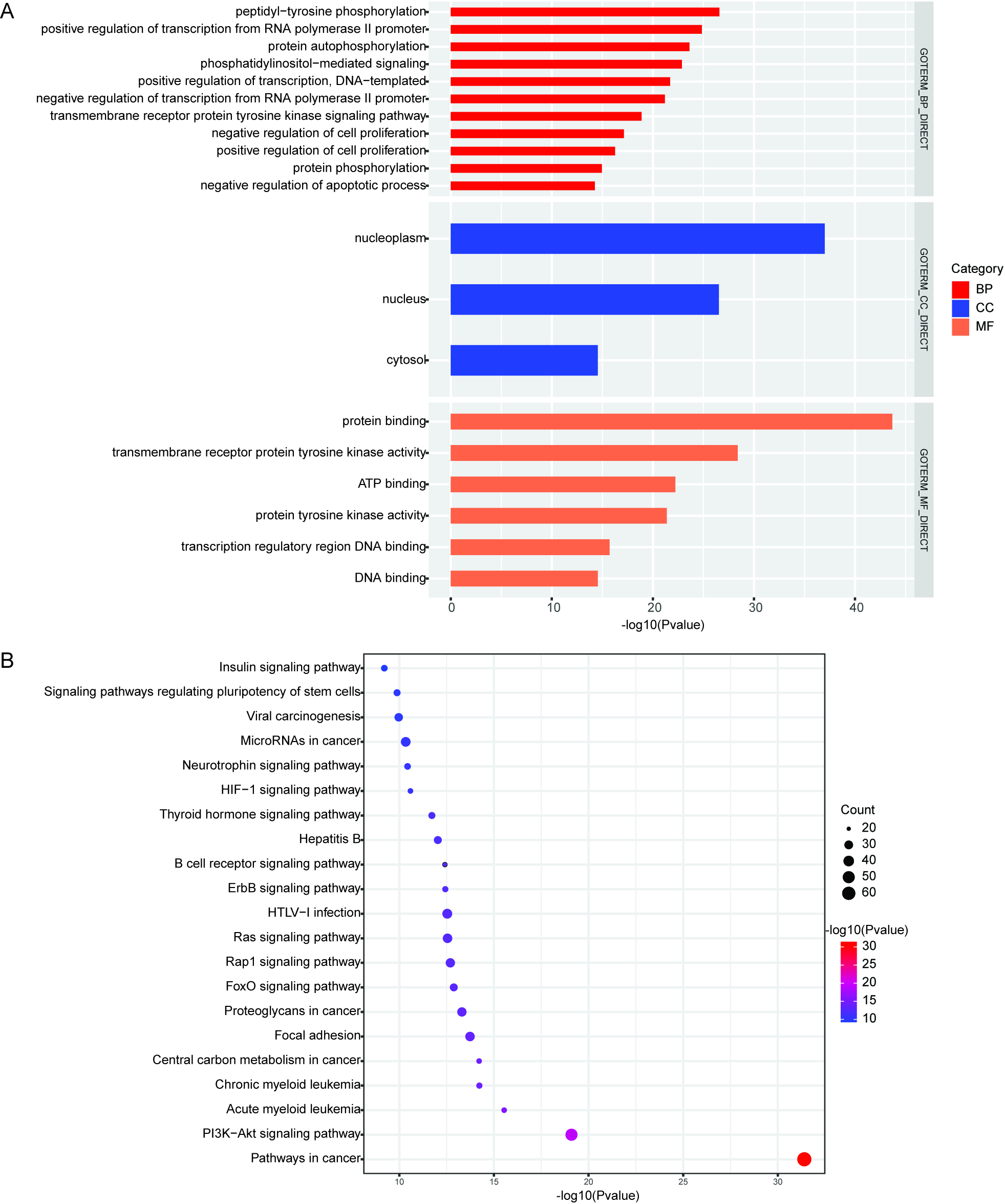

Supplement: Supplementary file 1 [file Image2.TIF]

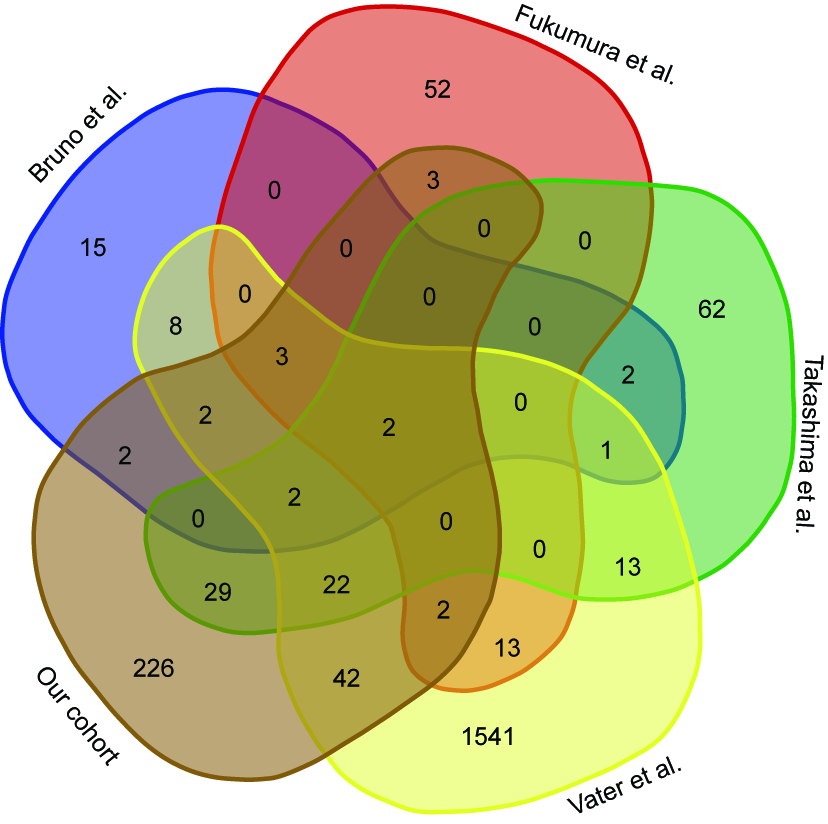

Supplement: Supplementary file 2 [file Image1.TIF]
